# Long non-coding RNA SNHG4 aggravates cigarette smoke-induced COPD by regulating miR-144-3p/EZH2 axis

**DOI:** 10.1186/s12890-023-02818-5

**Published:** 2023-12-19

**Authors:** Benyan Song, Yusi Chen

**Affiliations:** Department of Pulmonary and Critical Care Medicine, Affiliated Hospital of Panzhihua University, No. 27, Taoyuan Street, Bingcaogang, East District, Panzhihua, 617000 China

**Keywords:** Chronic Obstructive Pulmonary Disease, SNHG4, MiR-144-3p, EZH2, Inflammation

## Abstract

**Objective:**

The purpose of this study was to explore the expression level of SNHG4 in patients with COPD and its diagnostic value in COPD, to probe the biological function of SNHG4 in COPD at the cellular level, and to reveal the interaction between SNHG4 and miR-144-3p/EZH2 axis.

**Methods:**

The serum levels of SNHG4, miR-144-3p and EZH2 in healthy people and patients with COPD were detected by RT-qPCR. The diagnostic value of SNHG4 in COPD was evaluated by ROC curve. Pearson method was chosen to estimate the correlation between SNHG4 and clinical indicators in patients with COPD. Cigarette smoke extract (CSE) was obtained, and Beas-2B cells were exposed with 2% CSE to establish an inflammatory cell model of COPD in vitro. MTT assay was used to detect cell viability, flow cytometry was used to evaluate cell apoptosis, and ELISA was performed to detect inflammatory cytokines. Dual-luciferase reporting assay was carried out to verify the targeting of lncRNA-miRNA or miRNA-mRNA.

**Results:**

(1) The expression of SNHG4 is decreased in patients with COPD, and the expression level in acute exacerbation COPD was lower than that in stable COPD. SNHG4 demonstrated high diagnostic accuracy in distinguishing between stable and acute exacerbation COPD. (2) The expression of SNHG4 was decreased in CSE-induced Beas-2B cells, and overexpression of SNHG4 was beneficial to alleviate CSE-induced apoptosis and inflammation. (3) The expression of miR-144-3p is up-regulated in patients with COPD and CSE-induced Beas-2B cells. MiR-144-3p has a targeting relationship with SNHG4, which is negatively regulated by SNHG4. Overexpression of miR-144-3p could counteract the beneficial effects of increased SNHG4 on CSE-induced cells. (4) The expression of EZH2 is reduced in patients with COPD and CSE-induced Beas-2B cells. Bioinformatics analysis and luciferase reporter gene confirmed that EZH2 is the downstream target gene of miR-144-3p and is negatively regulated by miR-144-3p.

**Conclusion:**

The expression of SNHG4 decreased in patients with COPD, and it may promote the progression of COPD by inhibiting the viability, promoting apoptosis and inflammatory response of bronchial epithelial cells via regulating the miR-144-3p/EZH2 axis.

## Introduction

Chronic obstructive pulmonary disease (COPD) is a common chronic respiratory disease characterized by continuous airflow restriction, and its high morbidity and mortality have seriously threatened human health [1]. In the world, COPD is the fourth leading cause of death, and it is expected to become the third leading cause of death globally by 2030 [2]. Epidemiological investigation found that smoking is the most important factor causing COPD, especially in Western countries, 90% of patients with COPD have a history of smoking [3]. Active smoking and exposure to second-hand smoke can induce respiratory diseases, cardiovascular diseases and cancer, which not only affect the physical and mental health of individuals, but also cause huge economic losses to families and society. Although smoking has been recognized as an independent risk factor for COPD, the specific mechanism of smoking-induced COPD is still unknown, and effective preventive measures for smoking-induced lung injury are lacking. Therefore, it is of great theoretical significance to study the pathogenesis of smoking-induced COPD for the prevention and treatment of COPD.

Long non-coding RNA (lncRNA) consists of more than 200 nucleotides [4]. Due to their unique length and complex secondary and tertiary structure, lncRNA can function through interactions with proteins, RNA and DNA, and can influence gene expression in transcription, post-transcriptional modification and translation. Early researchers have observed changes in lncRNA expression in lung tissues of patients with COPD, such as PVT1 and linc00882 [5]. Chen et al. pointed out that the specific expression of HCG4B can be used as a possible therapeutic target for COPD [6]. These studies indicated that lncRNA is closely related to the occurrence of COPD. SNHG4 (small nucleolar RNA host gene 4) is a member of the SNHG family, located on chromosome 5q31.2. Studies have found abnormal expression of SNHG4 in many diseases, for example, decreased expression in diabetic retinopathy and acute myeloid leukemia [7, 8], while increased expression in endometriosis, gastric cancer, neuroblastoma and lung cancer [9–12]. In recent years, it has been found that SNHG4 silencing can aggravate lipopolysaccharide (LPS)-induced lung tissue inflammation in mice [13]. Considering the above studies and conclusions, we preliminarily speculate that SNHG4 may be abnormal in COPD.

MicroRNAs (miRNAs) are a class of highly conserved non-coding RNAs with a length of 22 nucleotides [14]. MiRNAs can interact with multiple genes at the same time and take part in some pathophysiological processes, such as cell differentiation, proliferation and immune response [15]. More and more evidence shows that abnormal miRNA is related to the occurrence of COPD, which may be a potential target for the treatment of COPD. For example, miR-125a-5p has been found to be elevated in smoking-induced COPD, and this miRNA exacerbates the disease by promoting the aging process of lung epithelial cells [16]. Another study showed that smoking can change the expression level of miRNA in bronchial epithelial cells and alveolar macrophages, leading to respiratory system damage [17]. MiR-144-3p is dysregulated in a variety of diseases. For example, the expression of miR-144-3p was enhanced in the group with major depression [18], but decreased in polycystic ovary syndrome [19]. Recently, miR-144-3p was reported to participate in the regulation of lung injury. Xu et al. reported that miR-144-3p is involved in the occurrence of acute lung injury in mice by regulating the JAK/STAT signaling pathway [20]. Therefore, it is speculated that miR-144-3p may also be involved in the regulation of COPD. Studies have shown that SNHG4 and miR-144-3p have binding sites, but it is still unclear whether SNHG4 can influence COPD by regulating miR-144-3p.

This study was aimed to investigate the expression levels of SNHG4 and miR-144-3p in the serum of clinical COPD subjects, and to explore the possible mechanism of SNHG4 and miR-144-3p in COPD through in vitro studies.

## Materials and methods

### Subjects

This protocol was supported by the Ethics Committee of Affiliated Hospital of Panzhihua University, and all subjects or their guardians signed informed consent. The subjects were from the outpatient department, inpatient department and physical examination centers of the respiratory department of Affiliated Hospital of Panzhihua University. The subjects included 50 patients with stable COPD, 50 patients with acute exacerbation COPD and 60 healthy controls. The inclusion criteria for acute exacerbation COPD were: (1) Consistent with the Chinese Guidelines for the Diagnosis and Treatment of Chronic obstructive Pulmonary Disease (Revised 2021) [21]; (2) Acute exacerbation stage: In the course of the disease, the condition deteriorates continuously beyond the daily situation, and the patient can have cough, shortness of breath or wheezing. In the short term, the sputum is purulent or mucous, and the inflammation such as fever can be significantly aggravated; (3) The heart, liver and kidney function are normal. The inclusion criteria for stable COPD were: (1) Consistent with the Chinese guidelines for the diagnosis and treatment of chronic obstructive pulmonary disease (Revised 2021); (2) The symptoms of cough, excessive phlegm and shortness of breath are stable or mild, and the symptoms are stable for no less than 3 months; (3) The heart, liver, and kidney function are normal. Exclusion criteria for patients with COPD: (1) Other acute or chronic diseases of respiratory system; (2) Hypertension, coronary heart disease, congestive heart failure, arrhythmia, septicemia, hematologic diseases, autoimmune diseases and malignant tumors; (3) Surgical treatment was performed within 6 months. Inclusion criteria of healthy control group: (1) Normal pulmonary function and chest imaging, no history of COPD or other respiratory diseases; (2) Normal heart, liver, and kidney function.

### Pulmonary function test and serum sample collection

The pulmonary function test is performed by a specialist in the pulmonary function room according to the standard pulmonary function test procedures. All patients were monitored using the pulmonary function instrument (MasterScreen PFT, Jaeger, Hochberg, Germany). The test is carried out in strict accordance with the general technical operating guidelines of the instrument. The calibration of temperature, humidity, pressure, test gas and volume were required before the measurement. The pulmonary function test was repeated three times for each patient, with an interval of 2 min for each test. The error of the three tests was less than 5%. Each patient inhaled 200 µg salbutamol, and after 15 min, the test was performed. Forced vital capacity (FVC) and forced expiratory volume in the first second (FEV_1_), FEV_1_/FVC, FEV1 as a percentage of the expected value (FEV1, predicted, %) were recorded for each patient.

On the next morning after enrollment, 5mL of fasting venous blood of each subject was collected, placed in an anticoagulant tube, centrifuged to separate the serum, and stored in a -80℃ refrigerator for subsequent experiments.

### Cell culture

In this study, Beas-2B cells were used for in vitro studies. Beas-2B cells were provided by American Type Culture Collection (ATCC) and cultured in DMEM medium containing 10% FBS and 1% penicillin/streptomycin in an incubator with adjustable humidity and 5%CO_2_ at 37 °C. Follow-up experiments were carried out with cells that had been passed to 3–5 generations and were in logarithmic growth phase.

### Preparation of cigarette smoke extract (CSE)

Referring to previous studies, 10 cigarettes were ignited, and the smoke bubbled through 30 mL of DMEM medium [22]. After the suspension was filtered and the pH was adjusted to 7.4, the solution is defined as the stock solution, i.e., 100% concentration. The stock solution was diluted to 0.5%, 1%, 2%, 5% and 10% (v/v) with DMEM medium and stored in the refrigerator at -80℃ for later use.

### Cell transfection

The expressions of SNHG4 or miR-144-3p were regulated by cell transfection techniques in vitro. SNHG4 expression vector was synthesized using pcDNA3.1 vector. MiR-144-3p mimic, miR-144-3p inhibitor and miR-negative control (miR-NC) were all provided by Gene Pharma (Shanghai, China). Lipofectamine 3000 (Invitrogen, Carlsbad, CA, USA) reagent was used for cell transfection. The specific operation procedure was carried out according to the kits’ instructions. In short, the transfection system is configured, which contains solution A and solution B. Solution A contains 125µL Opti-MEM and 5µL Lipofectamine 3000, and solution B contains 125µL Opti-MEM and 2.5µL plasmid. Solution A and solution B were mixed and added to the cells and continued to culture for 48 h. Subsequent operations were performed after transfection.

### RT-qPCR

TRIzol solution (Invitrogen, Carlsbad, CA, USA) was added to the serum samples, mixed, and left for 5 min, and then total RNA was extracted by chloroform, isopropyl alcohol and RNase-free ddH_2_O in sequence. An ultraviolet spectrophotometer was used to measure the RNA concentration and purity, and a value of A260/280 between 1.8 and 2.1 indicated that the extracted RNA was suitable for the experiment. The cDNA was synthesized using miScript kit (Qiagen, Germany). Then, PCR reaction was performed using the SYBR green PCR Master Mix kit (Life Technologies, Carlsbad, CA, USA) on 7500 real-time fluorescent quantitative PCR system. Reaction system: cDNA template (1µL), forward primer (0.5µL), downstream primer (0.5µL), SYBR Green (0.3µL), Taq HS Perfect Mix (10µL), ddH_2_O (7.7µL). Reaction conditions: pre-denaturation (94℃, 5 min), one cycle. Denaturation (94℃, 20s), annealing (60℃, 30s), extension (72℃, 40s), 40 cycles. Primers: SNHG4: (F) 5’-TTCAAGCGATTCTCGTGCC-3’; (R) 5’-AAGATTGTCAAACCCTCCCTGT-3; miR-144-3p: (F) 5’-ACACTCCAGCTGGGTACAGTAT-3’; (R) 5’-CTCAACTGGTGTCGTGGA-5’. GAPDH/U6 was used as the endogenous control for SNHG4/miR-144-3p. The relative expressions were analyzed by 2^−ΔΔCt^ method.

### Cell proliferation assay

Cell proliferation was assessed by MTT assay and counting cell numbers. Beas-2B cells of logarithmic growth stage were inoculated into 96-well plates at a density of 3 × 10^3^ cells/well, and cultured for 48 h. Cell groups were: Control group (conventional culture group), pcDNA group, pcDNA-SNHG4 group, CSE group (model group), CSE + pcDNA group, and CSE + pcDNA-SNHG4 group. After the cells were treated for 48 h according to the experimental procedure, MTT solution (5 mg/mL, Sigma-Aldrich, Waltham, MA, USA) was added, the supernatant was discarded after incubation for 4 h, and DMSO was added to the well and shaken sufficiently to dissolve the crystals. OD values at 490 nm wavelength were measured by microplate reader. After the cells were inoculated in the 96-well plate and performed according to the above experimental method, the cells were collected every 24 h and counted under the microscope with hematocytometer. Each experiment was repeated at least three times.

### Cell apoptosis assay

Beas-2B cells were inoculated into the 6-well plate at a density of 1 × 10^5^/mL. After the cells were treated according to the experimental procedure, cells were collected with trypsin and washed twice with PBS. 190 µL Annexin V-FITC binding fluid and 10 µL propidium iodide (PI) dye were added to the cells successively at room temperature and incubated for 10 min away from the light. Apoptosis was detected by flow cytometry (BD, Biosciences).

### ELISA

The cells of each group were collected, broken by repeated freezing and thawing, and the supernatant was collected by centrifugation at 3000r/min for 20 min. The concentrations of IL-1β, IL-8 and TNF-α in the cell supernatants of each group were detected according to the instructions of commercially available ELISA kit (Promega, Mannheim, Germany). Briefly, the standard product and the sample to be tested were added to the enzyme-labeled coated plate, followed by biotinylated anti-IL-1β, anti-IL-6 and anti-TNF-α antibodies, and incubated for 2 h. After cleaning the sample, horseradish peroxidase labeled avidin was added. Then, add color developer 100µL per well, and develop color at 37° for 15 min. Finally, the label plate was removed, and a termination solution was added to terminate the reaction (the color changes from blue to yellow immediately). The absorbance was measured at 450 nm by microplate reader, and the concentration of inflammatory factors was calculated according to the standard curve.

### Luciferase reporter gene

Target gene prediction of lncRNA was performed through starbase V 2.0 database. TargetScan, EVmiRNA and martarbase database were used to predict miRNA target genes. Luciferase reporter genes were used to verify the interaction between SNHG4 and miR-144-3p as well as the interaction between miR-144-3p and EZH2. In brief, Wild type SNHG4-3’UTR (WT-SNHG4) or wild type EZH2-3’UTR (WT-EZH2) were constructed by inserting the 3’UTR of SNHG4 or the 3’ UTR of EZH2 containing the complementary sequence of miR-144-3p into the luciferase reporter vector psi-CHECK. At the same time, mutant SNHG4-3’UTR (MUT-SNHG4) and mutant EZH2-3’UTR (MUT-EZH2) were constructed by site-specific mutation. Beas-2B cells were randomly divided into: (1) Control; (2) WT-SNHG4 (MUT-SNHG4) + miR-144-3p mimic; (3) WT-SNHG4 (MUT-SNHG4) + mimic-NC; (4) WT-SNHG4 (MUT-SNHG4) + miR-144-3p inhibitor; (5) WT-SNHG4 (MUT-SNHG4) + inhibitor-NC. Lipofectamine 3000 (Invitrogen, Carlsbad, CA, USA) was used to transfect the corresponding plasmid and miRNA into the cells, and the luciferase activity was detected by dual luciferase reporter gene system.

### Data analysis

SPSS 22.0 and GraphPad Prism 7.0 software were used for data analysis and image processing. Data conforming to normality is expressed as mean ± SD. The difference between groups was compared by independent sample t test, one-way ANOVA and Chi-square test. The receiver operator characteristic (ROC) curve was used to evaluate the clinical diagnostic value of SNHG4 in COPD. Pearson method was used to evaluate the correlation between miR-144-3p and inflammatory factors. *P* < 0.05 was considered to be significant difference. Each experiment was done in triplicate.

## Results

### Comparison of subjects’ baseline data

Baseline data for healthy controls and patients with COPD are summarized in Table 1. In this study, 100 patients with COPD were enrolled, including 50 patients with stable COPD and 50 patients with acute exacerbation COPD. Another 60 healthy people served as the control group in this study. The results showed that there is no significant differences in sex, age and body mass index (BMI) among the three groups (*P* > 0.05), so the three groups were comparable. It was also observed that the levels of FEV1/FVC, FEV1 (predicted) and inflammation cytokines (IL-1β, IL-6 and TNF-α) in patients with COPD were higher than those in healthy controls (*P* < 0.001). In addition, the levels of FEV1/FVC, FEV1 (predicted) and inflammation cytokines in patients with acute exacerbation COPD were higher than those in patients with stable COPD (*P* < 0.001).

**Table 1:** Comparison of baseline characteristics between COPD patients and healthy controls

Items	Control group (n = 60)	Stable COPD (n = 50)	Acute COPD (n = 50)	*P*
Age (years)	65.93 ± 5.72	66.29 ± 5.23	66.12 ± 5.65	0.247
Gender (male/female)	46/14	38/12	37/13	0.616
BMI (kg/m^2^)	22.89 ± 2.16	23.07 ± 1.98	22.75 ± 2.08	0.136
FEV1/FVC (%)	91.55 ± 3.01	61.28 ± 6.16	53.37 ± 4.15	< 0.001
FEV1 (predicted, %)	96.72 ± 3.24	69.79 ± 12.93	51.56 ± 14.33	< 0.001
Smoking history (n, %)	17 (28.33%)	24 (48.00%)	31 (62.00%)	0.009
IL-1β (pg/mL)	30.12 ± 11.38	58.74 ± 9.08	77.52 ± 8.46	< 0.001
IL-6 (pg/mL)	101.54 ± 41.77	149.33 ± 32.07	199.83 ± 37.84	< 0.001
TNF-α (pg/mL)	82.34 ± 25.83	140.26 ± 45.75	202.23 ± 60.15	< 0.001

Abbreviations: COPD, chronic obstructive pulmonary disease; BMI, body mass index; FEV1, forced expiratory volume in one second; FVC, forced vital capacity; IL-1β, interleukin-1β; TNF-α, tumor necrosis factor-α. *P* < 0.05 stands for significant difference

### The expression of SNHG4 and its clinical diagnostic value in COPD

**Fig. 1:**
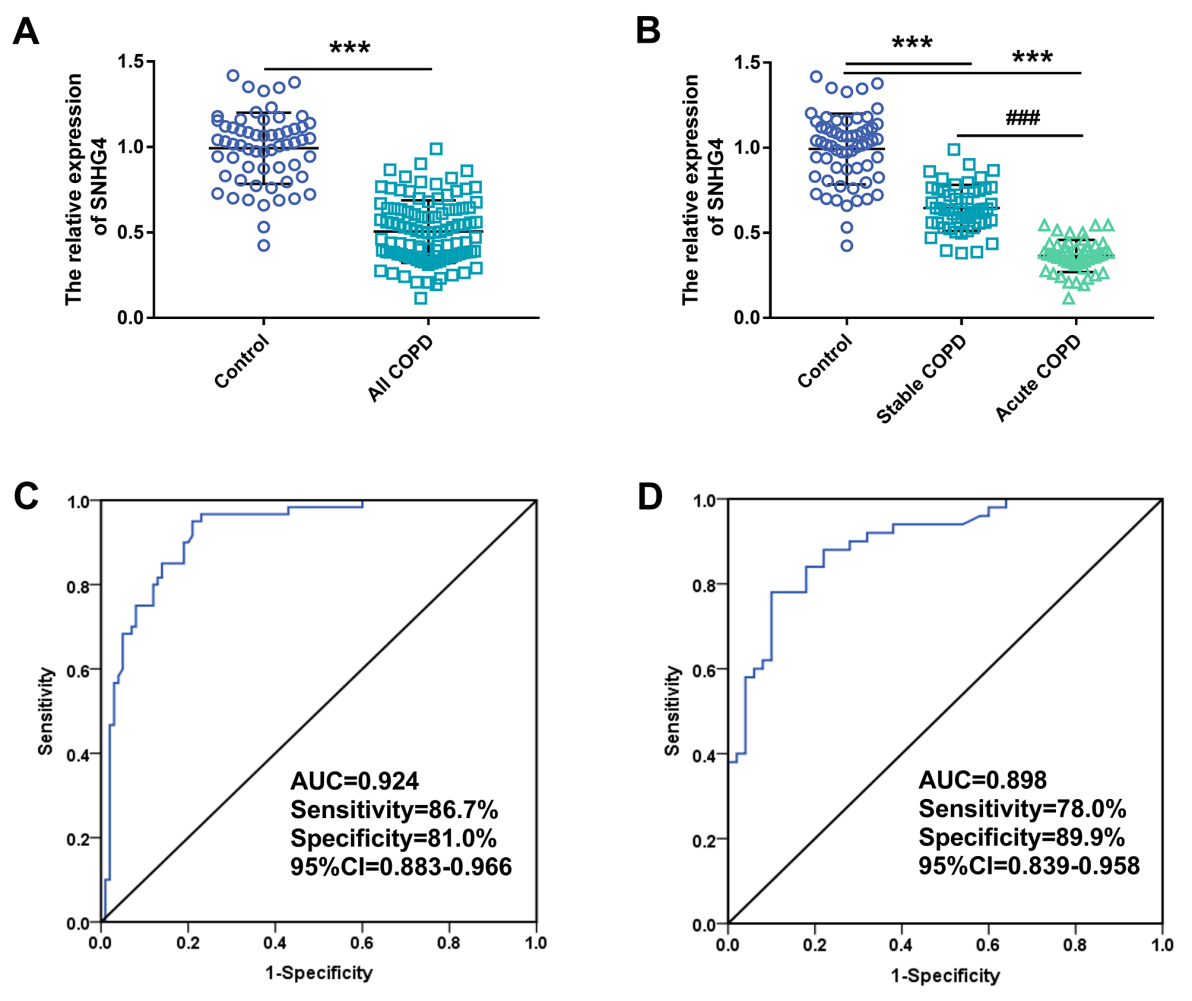
Expression of SNHG4 in COPD and its diagnostic value analysis. (**A**) Expression of SNHG4 in healthy people and patients with COPD. (**B**) Expression of SNHG4 in healthy people, stable COPD, and acute COPD. (**C**) The ability of SNHG4 to differentiate between healthy and COPD patients. (**D**) Diagnostic value of SNHG4 in patients with stable COPD and patients with acute COPD. ^***^P < 0.001, ^###^*P* < 0.001

RT-qPCR was used to detect the expression level of SNHG4 in serum. The results showed that compared with the healthy controls, the expression of SNHG4 in patients with COPD was significantly downregulated (*P* < 0.001, Fig. 1A). Patients with COPD were grouped into stable COPD and acute exacerbation COPD according to their conditions. Compared with the control group, the level of SNHG4 was reduced in both stable and acute exacerbation COPD, while the expression of SNHG4 in acute exacerbation COPD was lower than that in stable COPD (*P* < 0.001, Fig. 1B). Further, the clinical diagnostic efficacy of SNHG4 was evaluated by using ROC curve. Figure 1C showed the ability of SNHG4 to distinguish healthy people from COPD people. The AUC value of this curve is 0.924, and its sensitivity and specificity are 86.7% and 81.0%, respectively. Figure 1D showed the diagnostic ability of SNHG4 in stable COPD and acute exacerbation COPD, with an AUC of 0.898, sensitivity and specificity of 78.0% and 89.9%.

### Correlation between SNHG4 expression level and inflammatory cytokines in COPD patients

Pearson correlation coefficient method was operated to analyze the correlation between SNHG4 and inflammatory cytokines of patients with COPD, and the results were shown in Table 2. The results showed that SNHG4 was negatively correlated with the levels of IL-1β, IL-6 and TNF-α, indicating that the lower the level of SNHG4, the higher the concentration of inflammatory cytokines.

**Table 2:** Correlation between miR-144-3p and inflammatory markers

Inflammatory markers	coefficient (r)	*P*
IL-1β (pg/mL)	-0.568	< 0.001
IL-6 (pg/mL)	-0.642	< 0.001
TNF-α (pg/mL)	-0.609	< 0.001

Abbreviations: IL-1β, interleukin-1β; TNF-α, tumor necrosis factor-α

### Effect of SNHG4 on CSE-induced Beas-2B cells in vitro

**Fig. 2:**
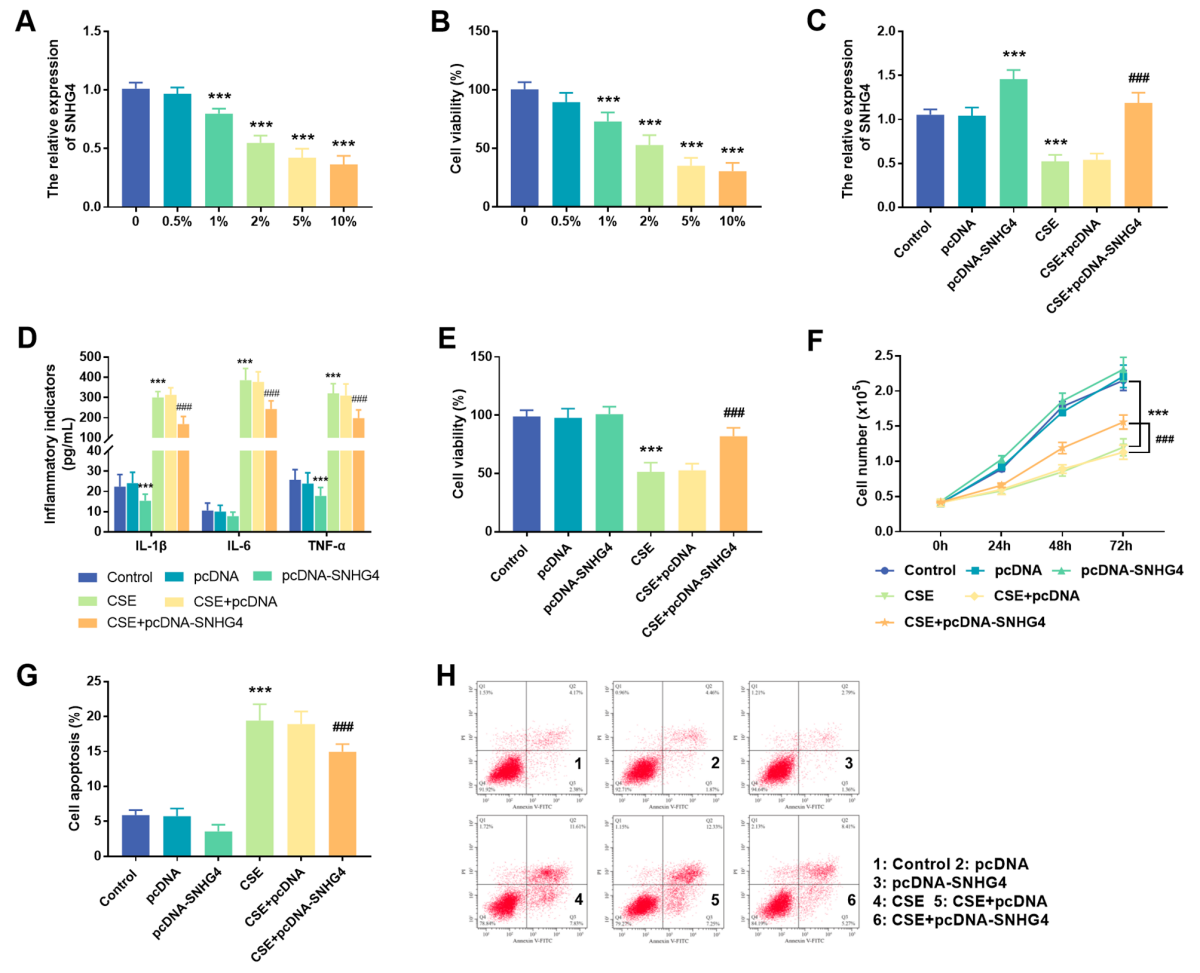
Effect of SNHG4 on Beas-2B cells. (**A**) The effect of different concentrations of cigarette extracts on SNHG4 expression in cells. (**B**) The effects of different concentrations of CSE on the viability of Beas-2B cells. (**C**) Cell transfection regulates intracellular SNHG4 expression level. (**D**) The effect of overexpression of SNHG4 on the inflammatory response of CSE-treated Beas-2B cells. (**E**) Effect of overexpression of SNHG4 on the viability of CSE-treated Beas-2B cells. (**F**) Effect of overexpression of SNHG4 on the proliferation number of CSE-induced Beas-2B cells. The effect of overexpression of SNHG4 on apoptosis of CSE-treated Beas-2B cells: (**G**) histogram and (**H**) scatter plot. ^***^P < 0.001, ^###^*P* < 0.001

In this study, Beas-2B cells were used as an in vitro cell model to study COPD. The results showed that the expression of SNHG4 in Beas-2B cells exposed to CSE gradually decreased with the increase of CSE concentration. When the CSE concentration was 2%, the level of SNHG4 decreased to 50%, so 2% was selected as the CSE exposure concentration in this study (*P* < 0.001, Fig. 2A). Similarly, the study also found that the viability of Beas-2B cells decreased with the increase of CSE concentration (*P* < 0.001, Fig. 2B). In order to investigate the potential biological function of SNHG4 in CSE-induced Beas-2B cells, intracellular expression of SNHG4 was modulated by in vitro cell transfection technique after exposure to 2% CSE. The results suggested that compared with conventional cultured cells, SNHG4 expression was decreased in CSE treated cells. However, after transfection with pcDNA-SNHG4, intracellular SNHG4 expression was up-regulated (*P* < 0.001, Fig. 2C). Next, the effect of SNHG4 on the CSE-induced inflammatory response was examined by ELISA. As expected, CSE induced an enhanced inflammatory response in Beas-2B cells, while overexpression of SNHG4 significantly down-regulated the levels of inflammatory cytokines, thereby inhibiting the inflammatory response (*P* < 0.001, Fig. 2D). Subsequently, the effects of SNHG4 on CSE-induced cell viability was detected by MTT assay and cell counting. AS shown in Fig. 2E-F, compared with conventional cultured cells (control group), the viability and cell numbers of CES exposed cells decreased (*P* < 0.001). Notably, after transfection of pcDNA-SNHG4, overexpression of SNHG4 reversed the inhibitory effect of CSE on cell proliferation (*P* < 0.001). Apoptosis was detected by flow cytometry. The results showed that late and early apoptotic cells increased significantly after CSE treatment, but the proportion of apoptotic cells was significantly reduced after increasing the expression level of SNHG4 (Fig. 2G-H, *P* < 0.001).

### Verification of the interaction between SNHG4 and miR-144-3p

**Fig. 3:**
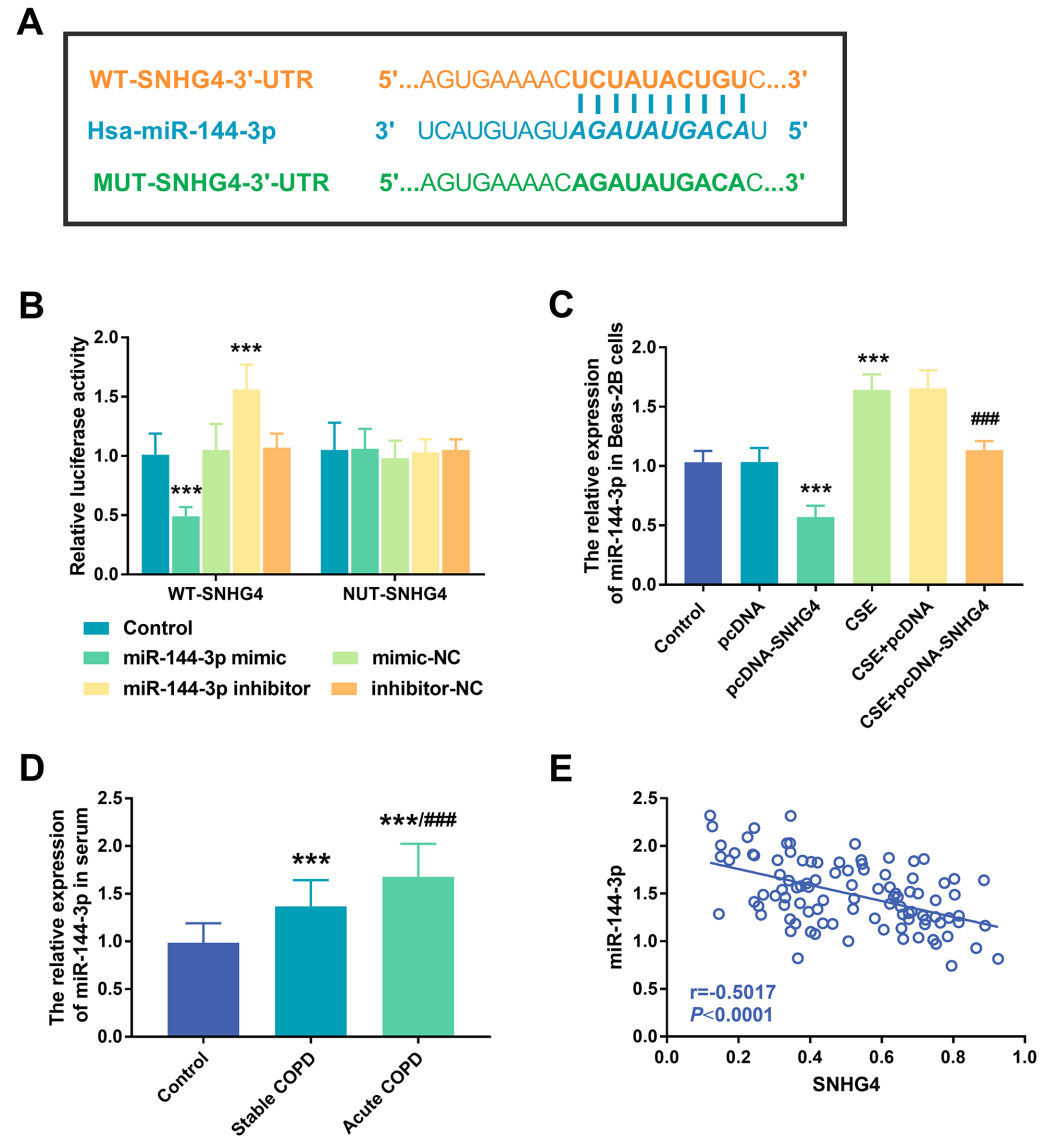
Verification of the interaction between SNHG4 and miR-144-3p. (**A**) SNHG4 and miR-144-3p complementary sequences. (**B**) Luciferase reporter gene analysis. (**C**) The effect of SNHG4-overexpression on miR-144-3p in Beas-2B cells. (**D**) The expression level of miR-144-3p in the serum of all the subjects. (**E**) Correlation between the expression of miR-144-3p and the expression level of SNHG4 in serum. ^***^P < 0.001, ^###^*P* < 0.001

Through Starbase V2.0 database, miR-144-3p and SNHG4 were proved to have complementary sites, and the sequences of these sites are shown in Fig. 3A. At the same time, we operated a dual-luciferase reporting gene assay, and found that the overexpression of miR-144-3p reduced the luciferase activity in WT-SNHG4 reporter vectors, but did not decrease luciferase activity in cells induced by MUT-SNHG4 reporter vectors (*P* < 0.001, Fig. 3B). Next, we measured the levels of miR-144-3p in the cells. Compared with conventionally cultured Beas-2B cells, the expression of miR-144-3p in Beas-2B cells exposed to CSE increased, while the expression of miR-144-3p was significantly inhibited by transfection of pcDNA-SNHG4 (*P* < 0.001, Fig. 3C). Similarly, the serum expression of miR-144-3p in the patients with COPD was opposite to that of SNHG4 in COPD. Compared with the control group, the expression of miR-144-3p in the serum of patients with COPD increased, and the level of miR-144-3p in patients with acute exacerbation COPD was apparently higher than that in patients with stable COPD (*P* < 0.001, Fig. 3D). Pearson analysis demonstrated that the serum expression level of miR-144-3p gradually decreased with the increase of the expression level of SNHG4, which indicated that the level of miR-144-3p was negatively correlated with the level of SNHG4 (*P* < 0.001, Fig. 3E).

### Effect of mir-144-3p on CSE-induced Beas-2B cells in vitro

**Fig. 4:**
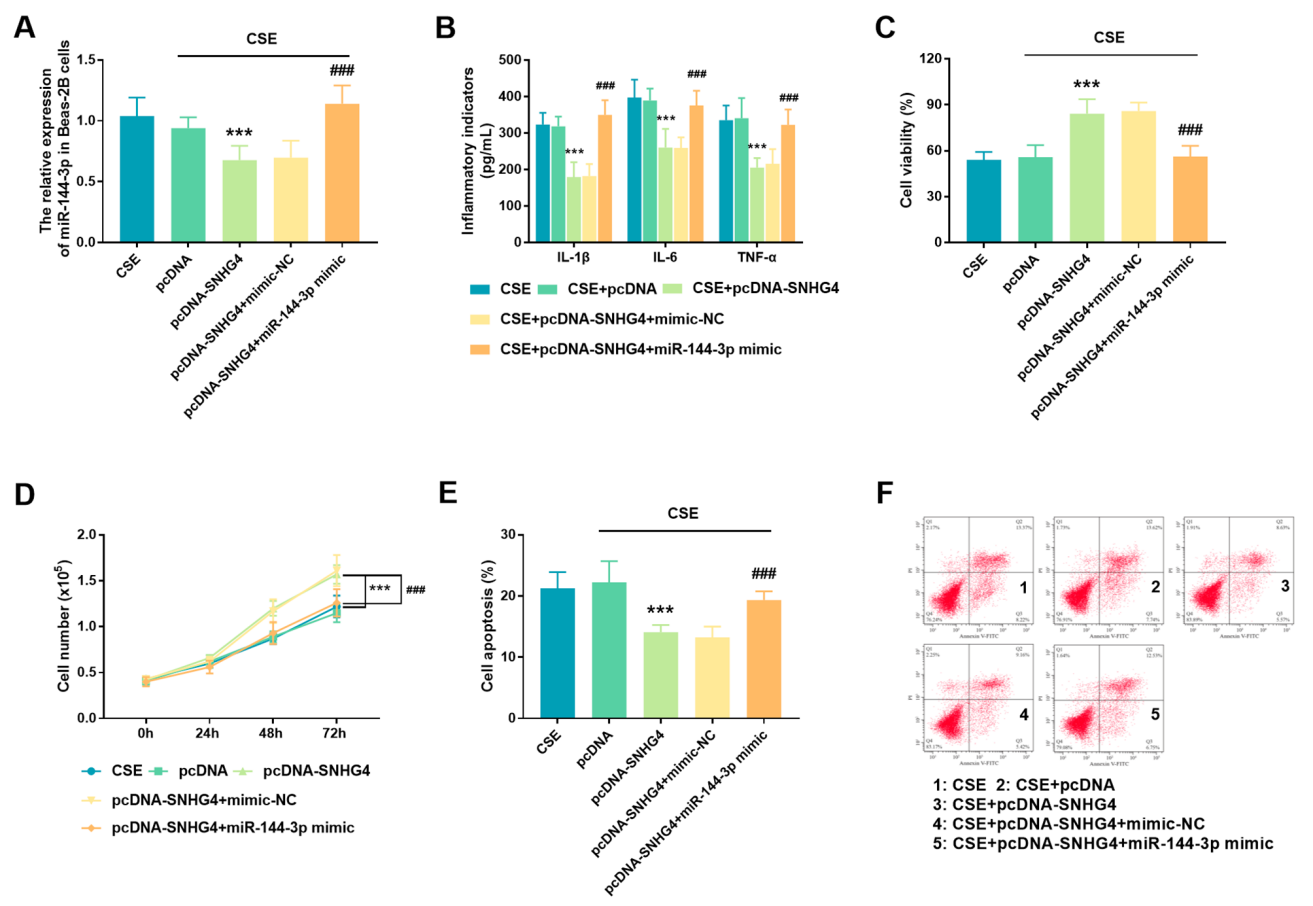
Effect of miR-144-3p on Beas-2B cells. (**A**) Cell transfection. (**B**) Effect of miR-144-3p overexpression on the inflammation response of CSE-treated Beas-2B cells. Effect of overexpression of miR-144-3p on (**C**) the viability and (**D**) proliferating cell count of CSE-treated Beas-2B cells. The effect of miR-144-3p on apoptosis was shown in (**E**) histogram and (**F**) scatter plot. ^***^P < 0.001, ^###^*P* < 0.001

To study the biological function of miR-144-3p in CSE-induced Beas-2B cells, the expression of miR-144-3p was modulated by transfecting miR-144-3p mimic or mimic NC. Results displayed that transfection of pcDNA-SNHG4 down-regulated the CSE-induced increase in miR-144-3p expression, however, this effect could be overturned by the presence of miR-144-3p mimic (*P* < 0.001, Fig. 4A). For cell inflammation, the increase of miR-144-3p promoted the production of inflammatory factors in Beas-2B cells (*P* < 0.001, Fig. 4B). For cell proliferation, the increase of miR-144-3p significantly inhibited cell viability and proliferating cell counts (*P* < 0.001, Fig. 4C-D). For cell apoptosis, overexpression of SNHG4 attenuated CSE-induced apoptosis, and this positive effect was also reversed by the increase of miR-144-3p (*P* < 0.001, Fig. 4E-F).

### Evidence of the interaction between mir-144-3p and its downstream target gene EZH2

**Fig. 5:**
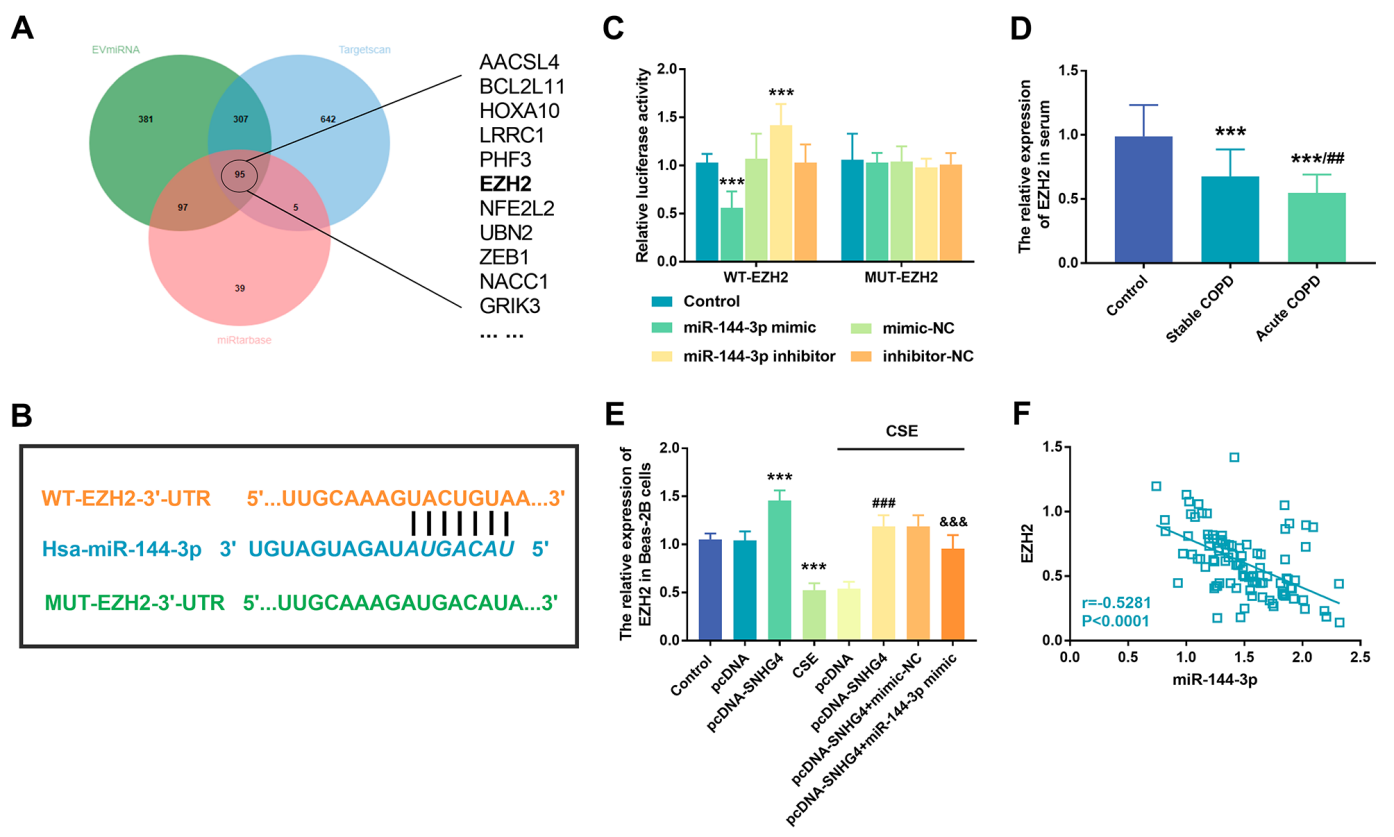
Verification of the interaction between miR-144-3p and EZH2. (**A**) Downstream target genes of miR-144-3p predicted by EVmiRNA and other databases. (**B**) Binding site of miR-144-3p and EZH2. (**C**) Luciferase reporter analysis. **(D**) The expression level of EZH2 in the serum of all the subjects. (**E**) The effect of miR-144-3p overexpression on EZH2 in Beas-2B cells. (**F**) Correlation between the expression of miR-144-3p and the expression of EZH2 in patients’ serum. ^***^P < 0.001, ^###^*P* < 0.001, ^&&&^*P* < 0.001

The Venn diagram composed of the three data is shown in Fig. 5A. The TargetScan, miRtarbase, and EVmiRNA databases together supported 95 downstream target genes of miR-144-3p, including EZH2. The complementary sequences of miR-144-3p and EZH2 are shown in Fig. 5B. Luciferase reporter gene assay exhibited that promotion or inhibition of miR-144-3p significantly weakened or enhanced luciferase activity in the WT-EZH2 reporter vector group but had no effect on luciferase activity in the MUT-EZH2 group (*P* < 0.001, Fig. 5C). In the serum samples of COPD patients, the expression of EZH2 in the acute exacerbation COPD group was lower than that in the stable COPD group (*P* < 0.001, Fig. 5D). In Beas-2B cells, EZH2 levels in CSE group was reduced compared with that in conventional culture group. The expression of EZH2 in SNHG4 overexpression group was increased. However, after further transfection of miR-144-3p mimic, the expression of EZH2 was reduced again (*P* < 0.001, Fig. 5E). Pearson analysis revealed that EZH2 levels were negatively correlated with miR-144-3p levels (*P* < 0.001, Fig. 5F).

## Discussion

The results of this study showed that: (1) the expression of SNHG4 was declined in COPD, and it was related to the severity of COPD. SNHG4 can distinguish between stable COPD and acute exacerbation COPD. (2) Upregulation of SNHG4 is helpful to alleviate the inhibition of CSE on Beas-2B cell viability, promote cell apoptosis and activate inflammatory response. (3) The expression of miR-144-3p was upregulated in COPD, which was negatively correlated with the expression of SNHG4. Overexpression of miR-144-3p can reduce cell viability and promote cell apoptosis and inflammation. (4) EZH2 is a downstream target gene of miR-144-3p, and it is negatively regulated by miR-144-3p. In this paper, we speculated that SNHG4 may play an unfavorable role in cell viability, apoptosis, and inflammation of CSE-induced Beas-2B by regulating the miR-144-3p/EZH2 axis.

LncRNA is widely involved in the occurrence and development of pulmonary physiological and pathological diseases, such as asthma, lung cancer, pulmonary fibrosis, etc., and plays an important role in cell inflammation and immunity [23, 24]. A microarray analysis reported the abnormal expression of 108 lncRNAs in lung tissue of mice in the cigarette exposure group and normal control group, and further animal analysis found that trachea inflammation was closely related to these abnormally expressed lncRNAs [25]. Previous literature studies have demonstrated the protective effect of SNHG4 in lung cancer and LPS-induced inflammatory lung injury, so we have reason to speculate that SNHG4 may be related to the occurrence of COPD. Based on this hypothesis, in this study, the expression of SNHG4 was found to be reduced in patients with COPD, and these patients were further divided into stable COPD and acute exacerbation COPD according to the severity of the disease. The expression of SNHG4 in patients with acute exacerbation COPD was lower than that in the stable COPD group, indicating that SNHG4 was associated with the severity of COPD. Furthermore, ROC analysis showed that SNHG4 had good clinical diagnostic accuracy in distinguishing stable COPD from acute exacerbations COPD.

The occurrence and development of COPD is a complex process. its pathogenesis involves inflammation, oxidative stress, and proteinase-antiprotease imbalance, but the specific molecular mechanism is still unclear [26]. In this study, Beas-2B cells were exposed to CSE to simulate the stimulation of cigarette smoke on bronchi, to construct a cell model of COPD in vitro and explore the molecular mechanism of SNHG4. The results showed that the expression of SNHG4 was significantly reduced in cells treated with 2% CSE, accompanied by decreased cell viability, increased apoptosis and increased secretion of inflammatory factors. In addition, by upregulating the level of SNHG4, the cell viability, apoptosis and inflammatory response were significantly improved. Currently, it is believed that miRNA, lncRNA and mRNA can affect disease occurrence through multi-site and multi-target mutual regulation [27]. This pattern of lncRNA-miRNA-mRNA regulates gene expression, which promotes the physiological and pathological processes, such as cell differentiation, proliferation and apoptosis. For example, in osteoarthritis, MALAT1 promotes inflammatory progression and extracellular matrix degradation by regulating the miR-150-5p/AKT3 axis [28]. Based on the sponge adsorption of lncRNA on miRNA, we conducted competitive endogenous RNA analysis to search for miRNAs that have a targeting relationship with SNHG4. This study found that miR-144-3p has a targeting relationship with SNHG4, and the former is negatively regulated by the latter. This relationship was supported by Zhou et al.’s study, who found an interaction and relationship between SNHG4 and miR-144-3p in colorectal cancer [29]. As previously reported, lncRNA RP11-86H7.1 can competitively inhibit the expression of miR-9-5p through the “sponge” effect, eliminate the inhibitory effect of miR-9-5p on NF-κB, and then up-regulates the expression of NF-κB1 and downstream inflammatory factors IL-6 and IL-8. Thus, trachea inflammation induced by traffic-related fine particulate matter was aggravated [30]. In recent years, the correlation between miR-144-3p and lung injury, inflammation, oxidative stress and immune response in COPD has been confirmed. In COPD, Wang et al. reported that exogenous LOC729178 alleviated the CSE-induced inflammatory damage of 16HBE cells by directly inhibiting the expression of miR-144-3p [31]. In Sepsis, NEAT1 inhibits the activation of NF-κB signaling pathway by up-regulating miR-144-3p, thus alleviating LPS-induced myocardial injury [32]. In this study, miR-144-3p can combine with SNHG4 and become its target miRNA. miR-144-3p is significantly up-regulated in both COPD patients and CSE-induced cells. The up-regulation of miR-144-3p can directly offset the beneficial effect of overexpression of SNHG4 on CSE-treated cells. This provides a possible therapeutic target for COPD.

MiRNAs can negatively regulate mRNA and inhibit translation processes, thus inhibiting protein output to an optimal level according to the cell type [33]. Combined with bioinformatics analysis and luciferase reporter gene experiments, we found that miR-144-3p could target EZH2 and negatively regulate the expression of EZH2 in this study. A decrease in EZH2 was observed in the blood of patients with COPD and in CSE-induced Beas-2B cells. Abnormal lung cell differentiation is a sign of many diseases, including COPD, and a report by Byrd et al. displayed that the deletion of EZH2 in mouse lung organoids weakened the self-renewal ability of cells and changes the progenitor cell population, leading to abnormal cell differentiation [34]. Snitow reported that EZH2 loss reduces the expression of myocardin and TBX18, thereby affecting the autonomic inhibition of smooth muscle differentiation in mesothelium cells, and ultimately contributing to the development of COPD and idiopathic pulmonary fibrosis [35]. Therefore, we inferred that miR-144-3p may facilitate CSE-induced apoptosis and inflammation in bronchial epithelial cells by inhibiting the expression of EZH2.

COPD is a multi-factorial disease with chronic inflammatory response as its main manifestation, in which the influence of genetic factors should not be underestimated [36]. In recent years, with in-depth studies of the regulatory network of lncRNA-miRNA-mRNA, the targeted regulatory relationship between COPD and downstream inflammatory signaling pathway has been gradually recognized. This provides a new idea for the pathogenesis and early diagnosis of COPD, and may provide a new breakthrough for the early diagnosis and intervention of COPD. Therefore, this study proposed that SNHG4 might regulate EZH2 by binding with miR-144-3p, affect the proliferation and apoptosis of bronchial epithelial cells in COPD, and activate the inflammatory response, thus participating in the pathogenesis of COPD. In recent years, there are more and more reports on non-coding RNA in respiratory diseases, but the role and mechanism of SNHG4 in regulating the miR-144-3p/EZH2 axis in COPD and bronchial epithelial cells have not been reported at home and abroad. This study will provide new ideas for the pathogenesis and early diagnosis of COPD, and may provide new breakthroughs for the early diagnosis and intervention of COPD.

Limitations of this study are as follows: Firstly, this was a single-center study with a small sample size. To further validate the expression of SNHG4 in COPD and evaluate its practical value as a diagnostic biomarker for COPD, extended studies in larger multicenter COPD cohorts are needed. Secondly, due to the limitation of experimental conditions, this study was not further verified in the cell and animal model of COPD. To explore the mechanism and function of SNHG4 in COPD and further explore the therapeutic effect of SNHG4 through in vitro and in vivo studies will provide theoretical basis for the prevention and treatment of COPD. There are still some inadequacies in the current research, and it is necessary to continue to expand the experiment in the future work.

## Data Availability

The datasets used and/or analysed during the current study are available from the corresponding author on reasonable request.
